# Object Affordances Tune Observers' Prior Expectations about Tool-Use Behaviors

**DOI:** 10.1371/journal.pone.0039629

**Published:** 2012-06-21

**Authors:** Pierre O. Jacquet, Valérian Chambon, Anna M. Borghi, Alessia Tessari

**Affiliations:** 1 Department of Psychology, University of Bologna, Bologna, Italy; 2 Institut National de la Santé et de la Recherche Médicale Unité 1028, Centre Nationale de la Recherche Scientifique UMR 5292, Lyon Neuroscience Research Center, ImpAct Team, Bron, France; 3 Centre National de la Recherche Scientifique UMR 5229, Centre de Neuroscience Cognitive, Université de Lyon, Bron, France; 4 Institute of Cognitive Sciences and Technologies, National Research Council, Rome, Italy; Royal Holloway, University of London, United Kingdom

## Abstract

Learning about the function and use of tools through observation requires the ability to exploit one's own knowledge derived from past experience. It also depends on the detection of low-level local cues that are rooted in the tool's perceptual properties. Best known as ‘affordances’, these cues generate biomechanical priors that constrain the number of possible motor acts that are likely to be performed on tools. The contribution of these biomechanical priors to the learning of tool-use behaviors is well supported. However, it is not yet clear if, and how, affordances interact with higher-order expectations that are generated from past experience – i.e. probabilistic exposure – to enable observational learning of tool use. To address this question we designed an action observation task in which participants were required to infer, under various conditions of visual uncertainty, the intentions of a demonstrator performing tool-use behaviors. Both the probability of observing the demonstrator achieving a particular tool function and the biomechanical optimality of the observed movement were varied. We demonstrate that biomechanical priors modulate the extent to which participants' predictions are influenced by probabilistically-induced prior expectations. Biomechanical and probabilistic priors have a cumulative effect when they ‘converge’ (in the case of a probabilistic bias assigned to optimal behaviors), or a mutually inhibitory effect when they actively ‘diverge’ (in the case of probabilistic bias assigned to suboptimal behaviors).

## Introduction

Tool-use refers to a type of behavior that consists in manipulating “external objects with the goal of altering the physical properties of another object, substance, surface, or medium, via a mechanical interaction”, or that consists in “mediating the flow of information between the tool user and the environment” ([1] pp.1203). A growing amount of evidence suggests that the acquisition of knowledge about object use and function through observation is not the privilege of human subjects [2]. Yet, the richness and complexity of our technology suggests that we are particularly well adapted for such competence [3]–[6]. It has been argued that this competence arises from a set of interpretative and learning predispositions that allows human observers to i) decode kinematic information into the causal relationships between a behavioral sequence and its result [7], ii) interpret biological movements performed by others as ‘rational’ (i.e. assuming that the most optimal actions means are adopted to achieve a particular goal) [8], and iii) accumulate knowledge from past observations about an agent's intentions and behaviors, and use this database in order to predict future events [9]–[13]. Together, these mechanisms would enable human observers to derive knowledge about the possible uses and functions of a tool from observing goal-directed, intentional movements performed by an agent [14]–[16]. In this article we posit that these sophisticated learning skills could also benefit from simpler heuristics allocated to the detection of low-level, local sources of information, such as the manipulative properties of objects [17].

These properties, called ‘affordances’, are not intrinsic to objects but depend on their possible interactions with agents [18]. In its extended form [19] an affordance defines a relational property that emerges from matching the perceived physical features of an object (e.g. size, shape, texture, density) and the agent's biomechanical architecture, her goals, plans, values, beliefs, and past experiences. They are also described as dispositional states of the agent's nervous system [20]. Critically, affordances ‘suggest’ how one may interact with an object [21], [22]. For example, the size and shape of a softball mean that it fits into the human hand, and its density and texture make it perfect for throwing. We posit that object affordances contribute to delineating the number of potential motor acts that can be performed on a given object. They do this by generating effector-dependent, biomechanical priors which are in line with the agent's bodily architecture [17]. These priors then bias individuals to act on objects with the aim of biomechanical optimization. In both human and non-human primates, preferentially performed behaviors are generally those that minimize the muscular and/or articulator costs, given the object's affordances and the desired outcome [23]–[26].

Crucially, this minimization of costs also transfers to tool use learning. A prominent example is provided by our extensive technologies. Humans deliberately manufacture tools whose complex physical attributes offer naïve users affordances that enable the extraction of their functions at low cost [27]–[29]. Interestingly, the evolution of human technology might have increased the utility of simple heuristics such as affordance detection, in order to facilitate the highly demanding cognitive problem of tool use learning [28], [30]–[32]. In our technological environments, the detection of affordances might thus play a crucial role in the acquisition of tool use skills through individual (i.e. trial-and-error learning) as well as social learning (i.e., learning from observing another agent's behaviors). Perceiving affordances may thus facilitate the extraction of functional features associated with an object manipulated by a third party [16]. For example, based on the amplitude of the observed agent's grip aperture and the orientation of her wrist, as well as on the size and texture of the object to be grasped, one may predict whether this object is meant to be lifted, pushed, or merely transported [11]. As suggested above, agents are expected to adopt tool-use behaviors that minimize biomechanical costs. Therefore, learning of a tool function through observation should be facilitated when a demonstrator uses a tool in a way that fit the observer's biomechanical expectations (behaviors that minimize the muscular and/or articulator costs), and should be jeopardized in the case where these expectations are patently violated (behaviors that increase the muscular and/or articulator costs).

Expert tool users, like tool learners, may also benefit from past experience in their daily interactions with objects [33]. It has been widely demonstrated that naïve human observers form knowledge (e.g. about tools and their potential use) by taking advantage of statistical regularities gathered from past observations [9]–[13]. The more times an individual associates a certain observed goal (e.g. the achieved tool function) with a certain observed action (e.g. the way of achieving the tool function), the more likely she is to expect that they will be seen together again [34]. These ‘probabilistic’ priors, acquired from past experiences, are crucial when the biomechanical information conveyed by tool affordances is too ambiguous or noisy to sufficiently constraint the range of candidate functions. Conversely, reference to biomechanical priors that are generated by tool affordances may be required when the use of the current tool cannot be based on previous experiences. Critically, both these classes of priors may be recruited when sensory information conveyed by movement kinematics is too incomplete to predict how an agent is most likely to behave. This occurs when many competing intentions are equally congruent with the not-yet completed behavior [11].

While the contribution of both these classes of priors to the individual-learning of tools' functions and use has long been demonstrated, it is not yet clear whether, and how, they may both combine to enable social learning of tool use (i.e., learning from observing another agent's behaviors). Here, we directly addressed this question in a task that required participants to predict, under various conditions of visual uncertainty, the intentions of a demonstrator who was using a multi-purpose tool. Affordance-related priors (termed ‘biomechanical’ priors) and priors acquired from past observations (termed ‘probabilistic’ priors) were manipulated by varying the biomechanical *optimality* of the tool behaviors and the *probability* (low versus high) of observing optimal versus suboptimal tool behavior.

We hypothesized that both biomechanical and probabilistic priors would have an effect on prediction. First, participants should be more accurate in predicting optimal than suboptimal behaviors (biomechanical bias). Second, participants should be more accurate in predicting behaviors that are most likely to occur throughout a specific experimental session (probabilistic bias). Third, we expected an interaction between these two classes of priors, whereby participants would preferentially respond towards the biased behaviors when the probabilistic bias is assigned to *optimal* behaviors. Finally, we expected this effect to vary as a function of the amount of visual uncertainty conveyed by the action being performed. Thus, the propensity to respond towards the biased behaviors should be strengthened as the amount of visual information shown in the action videos decreases.

## Methods

### Participants

Twenty-four healthy volunteers (mean age = 26.5, SD = 4.40) took part in an action prediction task. All were right-handed, naïve to the purpose of the experiment, and reported normal or corrected-to-normal visual acuity. The experimental protocol was performed with approval of the University of Bologna – Department of Psychology – ethical committee and in accordance with the Declaration of Helsinki (2008) [35]. All participants gave their verbal and informed consent to participate in the study. Owing to the non-invasive, purely behavioral nature of our study (without any emotional stimuli), the University of Bologna – Department of Psychology – ethical committee considered verbal consent was appropriate and approved this consent procedure. Socio-demographic information (full name, age, sex, gender, handedness, education) has been collected for each subject on a separate sheet. The sheet contained an “Approve” box that was checked by the experimenter after the subject gave their verbal consent to participate.

### Stimuli

**Figure 1:**
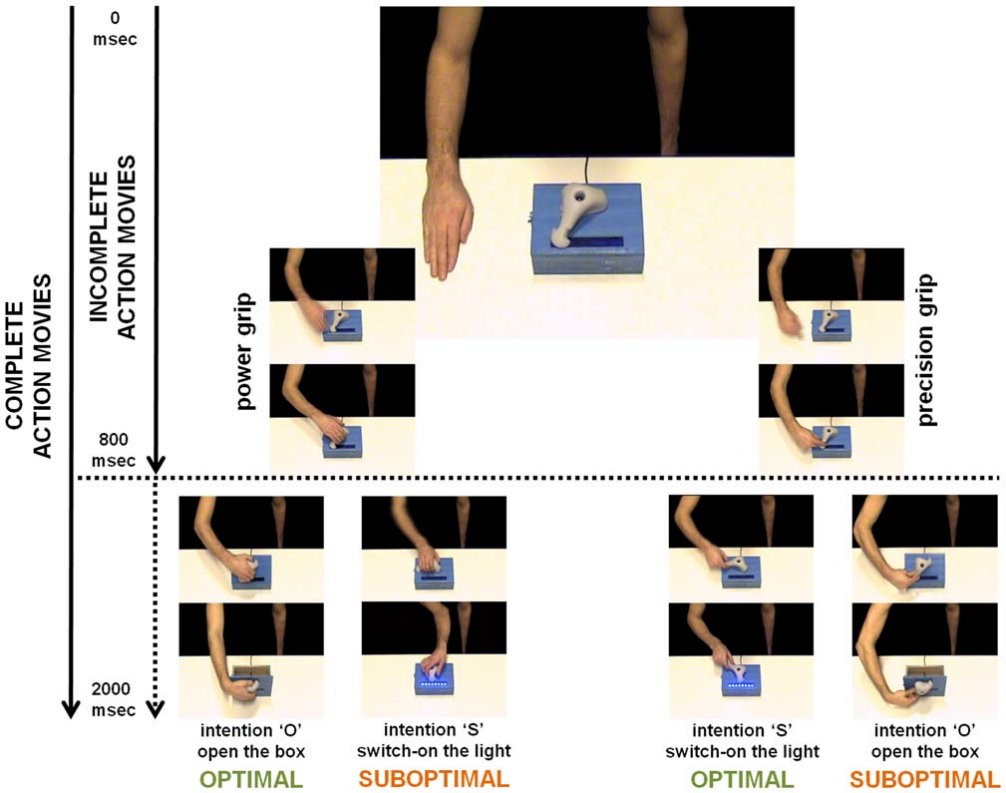
Examples of the four combinations ‘grip 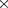 intention’ that participants encountered during the experiment, and that lead to ‘optimal’ or ‘suboptimal’ behaviors. All combinations began with the demonstrator's static hand. The actor could then use either a ‘power’ or a ‘precision’ grip to achieve either the intention of *Opening the box* (O) or *Switching the lights on* (S). The combination between the kind of grip and the kind of final intention resulted in the complete action as being labeled biomechanically optimal (OPTIMAL) or suboptimal (SUBOPTIMAL). Whereas the complete action movies lasted until the achievement of the underlying intention for a total duration of 2000 msec, the incomplete action movies stopped 800 msec after the movement onset (when the demonstrator was about to grasp the tool) while the last displayed frame remained on the screen for a duration of 1200 msec, so that observers had information about the grip but no information (on that trial) about the demonstrator's intention.

Stimuli consisted in movies featuring a demonstrator acting on a two-purpose tool. The tool consisted of a movable handle screwed onto the lid of a box. The handle offered two distinct affordances enabling the demonstrator to grasp the object with a power or a precision grip (see fig. 1). Using either grip, the demonstrator could achieve two intentions: *Opening the box by lifting the handle* (intention O); *Switching on the light by rotating the handle* (intention S) (see fig. 1).

Two movie formats were displayed, both having a total duration of 2000 msec (see fig. 1): a *complete* format in which actions lasted until the achievement of the underlying intention (the grasp and the demonstrator's final intention were apparent); an *incomplete* format in which action course stopped 800 msec after movement onset (only the grip was apparent but the demonstrator's final intention was not) while the last displayed frame was presented on the screen for the remaining 1200 msec.

All movies were equalized for temporal homogeneity in such a way that the duration of the sub-steps of each action involved the same number of video frames (sub-step 1: static hand to physical contact with the tool = 1000 ms; sub-step 2: physical contact with the tool to action end-state = 1000 ms).

### General Procedure

Participants sat in front of a monitor on which video clips that showed a male demonstrator acting on a tool were displayed (see fig. 1). The entire experiment was composed of three distinct experimental sessions. In each session, participants had a different probability of observing the demonstrator achieving his intentions using an *optimal* (cost-free) or a *suboptimal* (high cost) behavioral strategy [33].

For each of the three sessions, 4 blocks of 24 complete action movies were interleaved with 4 blocks of 12 incomplete action movies. Crucially, the probabilistic bias was exclusively assigned during the complete action movie blocks, where participants could benefit from a high amount of visual information to identify the demonstrator's intentions. In contrast, in the incomplete action movies the amount of visual information was too low for the observer to unambiguously infer the demonstrator's intention. Thus, blocks of complete action movies were used to generate prior expectations in favour of either the optimal or the suboptimal behavioral strategy. These expectations were induced through biased probabilistic exposure. In contrast, blocks of incomplete movies were used to test the effect of each type of bias (probabilistic and biomechanical biases) on the participants' decisions when confronted with visually uncertain action scenes (see [11], for a similar procedure).

For each of the 144 action movies, participants were required to predict the demonstrator's intention by pressing, with their right index and middle fingers, one of two adjacent computer keys corresponding to the two possible intentions. The procedure used was a self-paced procedure: participants were instructed to make their response as soon as they thought they had enough visual information to produce an accurate response. However, note that both complete and incomplete movies ran until completion independently of the subject's response.

### Typical trial

All trials started with a white fixation-cross that appeared for 1000 msec on a dark background. The fixation cross was immediately followed by either a complete or an incomplete action movie (see above for further details). After each decision, response time was displayed on the screen for 500 msec. For those trials in which participants did not respond, or responded too late, ‘NO RESPONSE’ was displayed on the screen. The next trial started immediately after the 500 msec visual feedback period. This feedback allowed us to avoid a ‘guessing bias’ that could occur during the presentation of complete action sequences, and that could hinder the integration of the probabilistic bias (see [11], for a similar procedure). The presentation of stimuli and recording of responses (correct/incorrect and response times) was synchronized using E-prime2 software (Psychology Software Tools, Inc, USA).

### Biomechanical priors

The four possible action combinations (2 grips 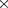
2 intentions) were divided into two types of behavioral category (optimal versus suboptimal) on the basis of their low or high biomechanical cost. This procedure allowed us to manipulate biomechanical priors emerging from perceived affordances (see fig. 1):

#### i) Optimal behaviors

Using the power grip to achieve the intention of opening the box by lifting the handle was cost-free, as was using the precision grip to achieve the intention of switching the lights on by turning the handle. These two combinations were identified as *optimal* behaviors (low biomechanical cost).

#### ii) Suboptimal behaviors

The precision grip increased the cost of achieving the intention of opening the box, whereas the power grip increased the cost of achieving the intention of switching on the lights. These two combinations were identified as *suboptimal* behaviors (high biomechanical cost).

The biomechanical cost of action movies were pre-tested on 10 naïve individuals. They were asked to estimate the muscular and/or articulator cost of each perceived movement on a 5-point Likert scale (ranging from 0 =  null cost to 5 =  very high cost). As expected, optimal behaviors (precision grip/switching-on the lights and power grip/opening the box, mean score  = 1.01) were estimated as significantly less costly than suboptimal ones (precision grip/opening the box and power grip/switchingsssssssss-on the lights, mean score  = 3.13) (two-tailed t-test for paired data: *t* = −20.87, p<.0001). It is of note that the intentions achieved with a precision grip were rated as less costly than those achieved with a power grip for both optimal (precision grip/switching-on the lights, mean score  = 0.55, versus power grip/opening the box, mean score  = 1.47; two-tailed t-test for paired data: *t* = −54.83, p<.0001) and suboptimal behaviors (precision grip/opening the box, mean score  = 2.90, versus power grip/switching-on the lights, mean score  = 3.37; two-tailed t-test for paired data: *t* = −30.82, p<.0001).

### Probabilistic priors

Unbeknownst to the participants, the probability of observing the demonstrator using an optimal or a suboptimal behavioral strategy was varied within the three distinct experimental sessions (‘*baseline’, ‘convergent bias’, ‘divergent bias*’ – see below). Varying the probability distributions of each possible strategy allowed us to manipulate each participant's probabilistic priors, that is, prior expectations they could form about the behavioral strategy being favored by the demonstrator to achieve the tool's functions. After each participant performed the task, we controlled for the extent to which she/he was aware of the induced bias. As expected, none of the subjects spontaneously reported that one type of action was more likely observed than another.

#### i)Baseline session: no probabilistic bias

In the first session, participants had an equal probability of observing the demonstrator achieving his intention by performing an optimal or a suboptimal behavior.

#### ii) ‘Convergent bias’ session: probabilistic bias towards optimal behaviors

In this session participants were biased towards ‘optimal’ behaviors to the detriment of ‘suboptimal’ behaviors. In 80% of the ‘box opening’ trials the demonstrator opened the box using a power grip, and in 80% of the ‘light switching’ trials he switched on the lights using a precision grip. Here, behaviors that were preferentially used by the demonstrator *converged* towards the participant's biomechanical priors.

#### iii) ‘Divergent bias’ session: probabilistic bias towards suboptimal behaviors

In this session participants were biased towards ‘suboptimal’ behaviors to the detriment of ‘optimal’ behaviors. In 80% of the ‘box opening’ trials the demonstrator opened the box using a precision grip, and in 80% of the ‘light switching’ trials he switched on the lights using a power grip. Here, the behaviors that were preferentially used by the demonstrator *diverged* from the participant's biomechanical priors.

All participants began the experiment with the baseline session. The order of the two bias sessions (convergent and divergent) was counterbalanced across participants.

### Training phase

Prior to the experiment participants were familiarised with the task. The training consisted of an unbiased complete action movie block followed by an incomplete action movie block.

### Data analysis

We analysed the percentage of correct responses (hits) and response times (RTs) collected for both complete and incomplete action movies. Responses for incomplete actions were encoded as correct if the predicted intentions conformed to those that the demonstrator actually achieved in their complete format. Participants who responded too early on more than 10 percent of the complete action movies were discarded from further analyses (responses were considered as too early when they occurred between 0 and 1000 msec after movie onset, making accurate predictions impossible). Using this criterion, two subjects were excluded.

All statistical analyses were performed separately for complete and incomplete action movies. The magnitude of the probabilistic bias and its interaction with biomechanical expectations was investigated by comparing performance during the baseline session with that during the two biased sessions. The hit rates and RTs were then analysed using a 2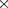 2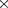 3 repeated-measures ANOVAs. The first two-level factor was the ‘type of behavior’ (optimal versus suboptimal behaviors), the second two-level factor was the ‘type of grip’ (power versus precision grip), and the third, three-level factor was the ‘probabilistic bias’ (baseline versus convergent bias versus divergent bias). Post-hoc Fisher tests were used to compare performance between conditions.

We further investigated the learning dynamics internal to each session by comparing data (hits and RTs) collected during the first (time-step 1) and the second half (time-step 2) of each session. Thus, for each session, the hits rates and RTs were analysed using 2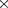 2 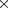 2 repeated-measures ANOVAs with ‘time-step’ (time-step 1 versus time-step 2), ‘type of behavior' (optimal versus suboptimal behaviors), and ‘type of grip’ (power versus precision grip) as two-level factors. Post-hoc Fisher tests were used to compare performance between conditions.

For all analyses, p<.05 was taken as the criterion for significance and eta squared (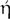) was used as a measure of effect size. Statistical analyses were performed using *Statistica 9* (www.statsoft.com).

## Results

### Overall performance

#### Complete action movies (Hits and RTs)

The 2 (type of behavior) 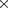
2 (type of grip) 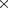
3 (probabilistic bias) repeated-measures ANOVAs revealed a main effect of the ‘type of behavior’ on both hits (F_1,21_ = 18.08, p<.001, 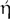 = .46) and RTs (F_1.21_ = 93.43, p<.0001, 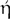 = .82). Participants were and faster at predicting optimal than suboptimal behaviors (hits: 88% vs. 81%; RTs: 1382 msec vs. 1444 msec). The main effect of the ‘probabilistic bias’ was also significant on both hits (F_2,42_ = 6.5, p<.01, 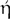 = .24) and RTs (F_2.42_ = 22.18, p<.0001, 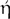 = .51). In the divergent bias session, participants made more accurate predictions compared to the baseline (hits: 88% vs. 84%, p<.05) and the convergent bias sessions (hits: 88% vs. 82%, p<.001). However, when compared to baseline, RTs were faster in both the convergent (1368 msec vs. 1452 msec, p<.0001) and the divergent bias sessions (1420 msec vs. 1452 msec, p<.05). It is of note that a difference occurred also between the two bias sessions, with faster RTs in the convergent bias session (1368 msec vs. 1420 msec, p<.001). Finally, a main effect of the ‘type of grip’ was found on hits only (F_1,21_ = 23.27, p<.0001, 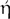 = .53), with participants being overall more accurate at predicting behaviors that were performed with a precision than a power grip (88% vs. 81%).

**Figure 2:**
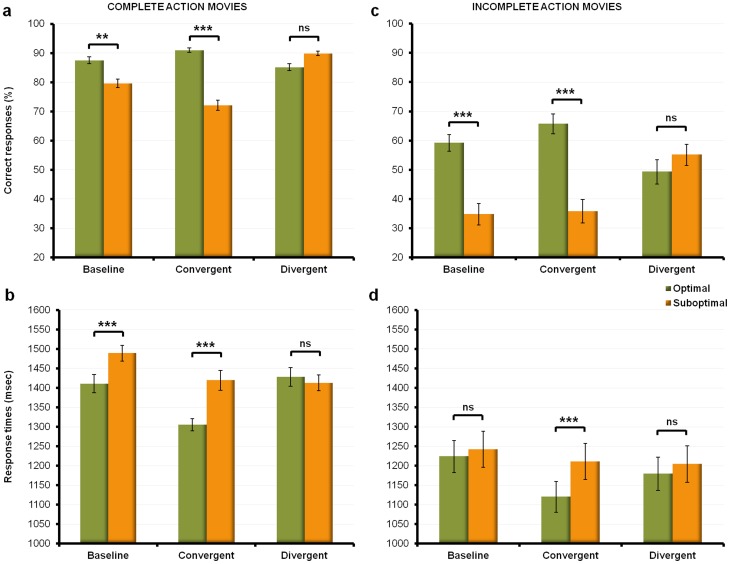
Overall performances. a) and c) represent the mean percentages of correct responses collected during complete and incomplete action movies for all three sessions. b) and d) represent the mean response times collected during complete and incomplete action movies for all three sessions. The green columns refer to the mean percentages of correct predictions for observed ‘optimal’ behaviors (pooled across ‘power’ and ‘precision’ grip). The orange columns refer to the mean percentages of correct predictions for observed ‘suboptimal’ behaviors (pooled across ‘power’ and ‘precision’ grip). Error bars denote the standard error of the mean.

The two-way interaction ‘type of behavior’ 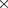 ‘probabilistic bias’ was significant for both hits (F_2.42_ = 19.76, p<.0001, 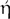 = .48) and RTs (F_2.42_ = 31.69, p<.0001, 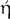 = .60) (see fig. 2a,b). Post-hoc comparisons (LSD Fisher tests) indicated that during the baseline session – where both types of behaviors were equally probable – participants were more accurate (87.5% vs. 80%, p<.01) and faster (1411 msec vs. 1492 msec, p<.0001) at predicting optimal compared to suboptimal behaviors. A similar pattern was observed in the convergent bias session. Participants were more accurate (91% vs. 72%, p<.0001) and faster (1308 msec vs. 1427 msec, p<.0001) at predicting the optimal behaviors when these behaviors were more frequently shown than the suboptimal ones. In the divergent bias session, no differences were found between the optimal and suboptimal behaviors, despite the fact that the latter were more frequently shown than the former (hits  = 85% vs. 90%, p>.05; RTs  = 1427 msec vs. 1414 msec, p>.05). Thus, increasing the probability of observing suboptimal behaviors did not significantly increase the number of correct responses for these behaviors compared to the optimal ones.

Interestingly, the interaction effect between the optimality of the behavior and the probabilistic bias was further modulated by the type of grip used, as revealed by a significant three-way interaction between all three factors for hits (F_2.42_ = 9.49, p<.001, 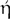 = .31). In the baseline session, the preference for optimal over suboptimal behaviors was observed for power grip only (post hoc test comparing optimal/power grip vs. suboptimal/power grip: p<.0001; post-hoc test comparing optimal/precision vs. suboptimal/precision grip: p>.05). In the convergent bias session, participants were impaired at predicting suboptimal over optimal behaviors irrespective of the type of grip used. In the divergent session, no difference between optimal and suboptimal behaviors was observed, irrespective of the type of grip used.

#### Incomplete action movies (Hits and RTs)

The 2 (type of behavior) 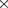
2 (type of grip) 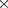
3 (probabilistic bias) repeated-measures ANOVAs revealed a main effect of the ‘type of behavior’ on both hits (F_1.21_ = 17.19, p<.001, 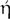 = .45) and RTs (F_1.21_ = 6.97, p = .01, 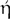 = .25); participants were more accurate and faster at predicting optimal than suboptimal behaviors (hits: 58% vs. 42%; RTs: 1176 msec vs. 1215 msec). This preference for optimal behaviors significantly differed from chance (t-test for single mean compared to 50, t>4.40, p<.001).The main effect of the ‘probabilistic bias’ was significant only for RTs (F_2,42_ = 5.75, p<.01, 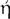 = .21). This indicated that, compared to the incomplete movie blocks of the baseline session, participants make faster predictions in the incomplete movie blocks of the convergent bias (1156 msec vs. 1235 msec, p<.01). Note that they also tended to make faster predictions in the incomplete movies of the divergent bias session (1194 msec vs. 1235 msec, p = .08). The main effect of ‘type of grip’ was not significant (hits and RTs: all F>.33, all p>.48).

The two-way interaction ‘type of behavior’ 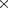 ‘probabilistic bias’ was significant for both hits (F_2,42_ = 9.84, p<.001, 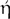 = .32) and RTs (F_2,42_ = 3.34, p<.05, 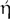 = .14) (see fig. 2c,d). As for the complete movie blocks, post-hoc comparisons (LSD Fisher tests) indicated that, in the baseline session, participants were more accurate at predicting optimal than suboptimal behaviors (59% vs. 35%, p<.001). This preference for optimal behaviors significantly differed from chance (t-test for single mean compared to 50, t>3.32, p<.01).They were also more accurate (66% vs. 36%, p<.0001) and faster (116 msec vs. 1197 msec, p<.001) at predicting optimal than suboptimal behaviors in the incomplete action movie blocks of the convergent bias session. Again, the preference for optimal behaviors was significantly different from chance level (t-test for single mean compared to 50, t>4.75, p<.001). However, in the incomplete action movie blocks of the divergent bias session, we did not find any differences between the optimal and the suboptimal behaviors, although the latter were most likely observed than the former in the complete movie blocks that preceded (hits  = 49% vs. 55%, p>.05; RTs  = 1187 msec vs. 1202 msec, p>.05). Note that performances for both optimal (t-test for single mean compared to 50, t<−0.17, p>.05) and suboptimal behaviors (t-test for single mean compared to 50, t>1.46, p = .15) did not significantly differ from chance.

Finally, the interaction effect between the ‘type of behavior’ performed (optimal vs. suboptimal) and the ‘probabilistic bias’ (baseline vs. convergent vs. divergent) was modulated by the type of grip (power vs. precision) used by the demonstrator (F_2,42_ = 3.37, p<.05, 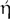 = .14). In the incomplete action movie blocks of the baseline and convergent bias sessions, the difference between optimal and suboptimal behaviors was observed independently of the type of grip used. In the incomplete action movie blocks of the divergent bias session, a difference between optimal and suboptimal behaviors was observed only when both of them were achieved by a precision grip (optimal/precision  = 47% vs. suboptimal/precision  = 59%). Note that the proportion of correct predictions for suboptimal behaviors achieved with a precision grip differed from chance (t-test for single mean compared to 50, t>2.38, p<.05).

#### Overall performance: preliminary discussion (fig. 2)

Results for the complete action movies demonstrate that, compared to baseline, the probabilistic bias significantly improved participants' performance – as also indicated by faster reaction times in the two bias sessions. Note that the rate of correct responses was overall higher in the divergent session. This is easily explained by the fact that, in the convergent session, the probabilistic bias assigned to optimal behaviors concomitantly increased the errors rate for unbiased (i.e., suboptimal) behaviors. In contrast, the probabilistic bias assigned to suboptimal behaviors did not alter the participants' ability to accurately predict the unbiased (i.e., optimal) behaviors. Thus, the higher the probability that a behavior occured, the better and faster it was predicted, irrespective of its type (optimal or suboptimal). These results indicate that, as expected, participants were successful in integrating the probability distributions of both convergent and divergent bias sessions.

The second set of results shows that the biomechanical constraints generated by the detection of tool affordances play a major role in participants' predictions: participants were more accurate and faster at predicting behaviors that minimized biomechanical costs, irrespective of probabilities. Thus, in both the complete and incomplete action movies of the baseline session (i.e. a session in which the demonstrator equally selected between the two available behavioral strategies), participants preferentially chose intentions achieved by optimal behaviors rather than suboptimal behaviors (see fig. 2a,b,c,d). This result demonstrates that when participants cannot rely on past observations (i.e., on probability) to decide how an observed agent is most likely to behave, they tend to rely on their biomechanical priors *by default*. That is, they assume that the observed agent behaves ‘rationally’, i.e., that he favors strategies which minimize biomechanical costs.

The third set of results concerns the interaction between the two kinds of priors (biomechanical and probabilistic) (fig. 2a,b,c,d). We found that both the magnitude and dynamics of the probabilistic bias differed as a function of the type of behavior, with participants' biomechanical expectations overriding the effect of the probabilistic bias. Thus, in the convergent bias session (probabilistic bias assigned to optimal behaviors) performance decreased for the suboptimal behaviors, and was facilitated for the optimal behaviors, as expected. This pattern of performance – observed in both the incomplete movie and complete movie blocks – suggests that it is costly for participants to inhibit a response that fits with their biomechanical expectations, even though a high amount of visual information is available. However, in the divergent bias session (probabilistic bias assigned to suboptimal behaviors), no significant differences were found between the two alternatives: participants did not preferentially choose the suboptimal behavior over the optimal one, although the former was more likely to be performed than the latter. This pattern suggests that participants actively integrated both types of priors, by combining their respective effects. Thus, when probabilistic and biomechanical priors diverged, the overall effect tended to sum to zero, resulting in performances that did not significantly differ from chance for both optimal and suboptimal behaviors.

Finally, we found that the type of grip used by the demonstrator had an effect on the participants' predictions when i) the probability of each competing intention was equal (baseline session), and ii) when the intention that was eventually achieved was fully visible (complete movies). This finding can be accounted for by a *facilitatory effect* of the precision grip. Although suboptimal behaviors that were achieved with a precision grip were estimated as suboptimal, they were nevertheless estimated as less constraining than those performed with a power grip. Interestingly, this facilitatory effect was easily overcome by the probabilistic bias, since it disappeared in both the convergent and divergent bias sessions. It is of note that this tendency to over-estimate the optimality of precision grips may be due to the biomechanical characteristics of the effector itself. Indeed, performing prehension movements with either a power grip or a precision grip differentially affects the synergies of arm segments. While the achievement of a power grip exerts constraints on many degrees of freedom of the arm (i.e. the wrist, elbow and shoulder) [36], the precision grip offers more flexible solutions [37], independently of the overall cost of the final action (e.g. opening the box with a precision grip).

### Learning dynamics

#### Complete action movies (Hits and RTs)

**Figure 3:**
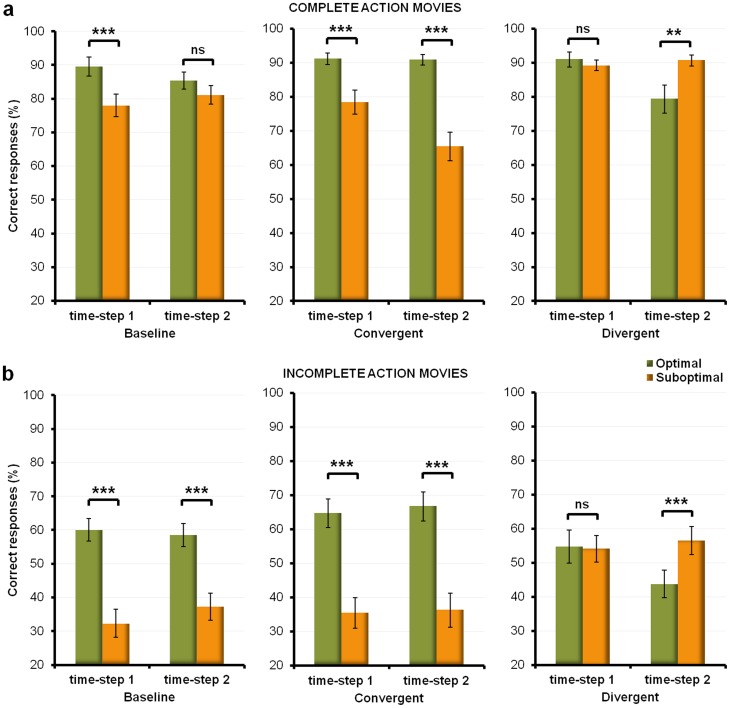
Learning dynamics. a) and b) represent the mean percentages of correct responses collected during complete and incomplete action movies for all three sessions. The green columns refer to the mean percentages of correct predictions for ‘optimal’ behaviors (pooled across ‘power’ and ‘precision’ grip). The orange columns refer to the mean percentages of correct predictions for ‘suboptimal’ behaviors (pooled across ‘power’ and ‘precision’ grip). Error bars denote the standard error of mean.

*i) Baseline session*. The 2
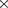
2
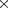
2 repeated-measures ANOVA performed on ‘time-step’ (time-step 1 vs. time-step 2),‘type of behavior’ (optimal vs. suboptimal) and ‘type of grip’ (power vs. precision grip) revealed a main effect of the ‘type of behavior’ for both hits (F_1,21_ = 11.57, p<.01, 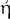 = .36) and RTs (F_1,21_ = 47.7, p<.0001, 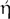 = .69), with optimal behaviors being overall faster (1411 msec vs. 1493 msec) and more accurately predicted (88% vs. 80%) than suboptimal ones. A main effect of ‘type of grip’ was also found on hits only (F_1,21_ = 9.48, p<.01, 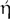 = .31), with behaviors achieved using a precision grip being overall more accurately predicted than those achieved using a power grip (87% vs. 80%). The two-way interaction ‘time-step’ 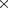 ‘type of behavior’ was significant for hits (F_1,21_ = 4.91; p<.05, 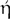 = .19) (see fig. 3a). Post-hoc comparison tests (LSD Fisher tests) showed that the difference between the percentage of hits observed at time-step 1 for the optimal and the suboptimal behaviors (90% vs. 78%; post-hoc test: p<.0001) was no longer significant at time-step 2 (85% vs. 82%; post-hoc test: p>.05). Neither the main effect of ‘time-step’, nor the two-way interaction ‘time-step’ 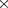 ‘type of grip’, nor the three-way interaction was significant (hits and RTs: all F<2.93, all p>.10).

*ii) Convergent bias session*. The same 2
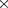
2
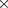
2 repeated-measures ANOVA performed on complete movie blocks of the convergent bias session revealed main effects of ‘time-step’ (hits: F_1,21_ = 9.80; p<.01, 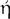 = .32; RTs: F_1,21_ = 6.87; p<.05, 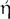 = .25) and ‘type of behavior’ (hits:_ F1,21_ = 34.09; p<.0001, 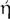 = .62; RTs: F_1,21_ = 43.61; p<.0001, 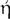 = .67) on both hits and RTs. Participants were more accurate but slower at predicting the demonstrator's intention at time-step 1 than at time-step 2 (hits  = 85% vs. 78%, p<.01); RTs  = 1386 msec vs. 1337 msec, p<.05). Overall, they were more accurate and faster at predicting likely optimal than unlikely suboptimal behaviors (hits  = 91% vs. 73%, p<.0001; RTs = 1307 msec vs. 1416 msec, p<.0001). A main effect of the ‘type of grip’ was also shown on hits only (F_1,21_ = 17.26; p<.001, 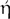 = .45), revealing that participants more accurately predicted behaviors performed with a precision than a power grip (87%vs. 77%, p<.001), independently of their optimality and of the time-step. Furthermore, the two-way interaction ‘time-step’ 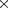 ‘type of behavior’ was significant for hits (F_1,21_ = 9.07; p<.01, 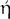 = .30) (see fig. 3a). Post-hoc analyses (LSD Fisher tests) showed that throughout the session, participants were overall more accurate at predicting the optimal than the suboptimal behaviors, and that this advantage for optimal behaviors increased over time (time-step 1 = 91% vs. 79%, p<.001; time-step 2 = 91% vs. 66%, p<.0001). The two-way interaction between ‘time-step’ 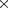 ‘type of grip’ as well as the three-way interaction were not significant (hits and RTs: all F<1.60, all p>.22).

*iii) Divergent bias session*. The same 2
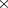
2
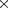
2 repeated-measures ANOVA performed on complete movie blocks of the divergent bias session showed a main effect of ‘time-step’ (F_1,21_ = 5.04.; p<.05, 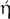 = .19), with better performance at time-step 1 than at time-step 2 (90% vs. 85%). A main effect of the ‘type of grip’ was also found on hits (F_1,21_ = 6.99.; p<.05, 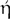 = .25), with better performance for behaviors performed with a precision than a power grip (90% vs. 84%), irrespective of their optimality. The interaction between the ‘time-step’ and ‘type of behavior’ factors was significant for hits only (F_1,21_ = 6.85.; p<.05, 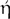 = .25) (see fig. 3a). In the first half of the session participants performed equally well (post-hoc test: p>.05) for the likely suboptimal (time-step 1 = 89%) and the unlikely optimal behaviors (time-step 1 = 91%). In the second half, however, they were more accurate at predicting the suboptimal behaviors (time-step 2 = 91% vs 79%; p<.01). This was associated with decreased performance for the unlikely optimal behaviors throughout the session (time-step 1 = 90% vs. time-step 2 = 79%). The main effect of ‘type of behavior’, the ‘time-step’ 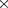 ‘type of grip’ interaction, and the three-way interaction were not significant (hits and RTs: all F<3.83, all p>.06).

#### Incomplete action movies (Hits and RTs)

*i) Baseline session*. The 2
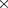
2
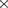
2 repeated-measures ANOVA performed on ‘time-step’ (time-step 1 vs. time-step 2),‘type of behavior’ (optimal vs. suboptimal) and ‘type of grip’ (power vs. precision grip) showed a main effect of the ‘type of behavior’ on hits only (F_1,21_ = 17.96, p<.001, 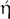 = .46). In the incomplete movie blocks of the baseline session, participants were more accurate at predicting optimal (59%) than suboptimal (35%) behaviors, independently of the time-step. Neither the main effects of ‘time-step’ or ‘type of grip’, nor the two-way interactions ‘time-step’ 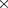 ‘type of grip’ and ‘time-step’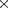 ‘type of behavior’ (see fig. 3b), nor the three-way interaction were significant (hits and RTs: all F<1.21, all p>.28).

*ii) Convergent bias session*. The same 2
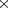
2
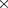
2 repeated-measures ANOVA performed on incomplete movie blocks of the convergent bias session revealed a main effect of ‘time-step’ on RTs only (F_1,21_ = 9.53; p<.01, 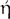 = .31). Overall, participants responded slower at time-step 1 (1178 msec) than at time-step 2 (1141 msec). A main effect of the ‘type of behavior’ was present for both RTs (F_1,21_ = 14.11; p<.01, 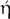 = .40) and hits (F_1,21_ = 21.17; p<.001, 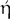 = .50), with participants being more accurate (66% vs. 36%) and faster (1116 msec vs. 1203 msec) at predicting optimal than suboptimal behaviors. The main effect of the ‘type of grip’, the ‘time-step’ 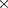 ‘type of grip’ and ‘time-step’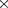 ‘type of behavior’ interactions (see fig. 3b), and the three-way interaction were not significant (hits and RTs: all F<3.77, all p>.07).

*iii) Divergent bias session.* The same 2
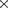
2
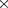
2 repeated-measures ANOVA performed on incomplete movie blocks of the divergent bias session showed a significant interaction between the ‘time-step’ and ‘type of behavior’ on hits only (F_1,21_ = 8.39; p<.01, 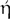 = .27) (see fig. 3b). Post-hoc tests (LSD Fisher tests) demonstrated that in the first half of the incomplete movie blocks, rates of correct predictions for the optimal and the suboptimal behaviors did not differ (time-step 1 = 54% vs. 54%; p>.05). However, a difference occurred in the second half of the incomplete movie blocks, with suboptimal behaviors being more accurately predicted than optimal ones (time-step 2: optimal 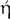 = 44% vs. suboptimal 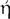 = 57%; p<.001). Of note is the fact that this effect was due to the rate of correct predictions for the optimal behaviors decreasing over the session (time-step 1 = 54% vs. time-step 2 = 44%; p<.01). However, neither the performance for suboptimal behaviors (t-test for single mean compared to 50, t<1.47, p = .15) nor the performance for optimal behaviors (t-test for single mean compared to 50, t<−1.32, p = .19) significantly differed from chance level. No significant main effects were revealed (hits and RTs: all F<1.87, all p>.19). Neither the ‘time-step’ 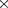 ‘type of grip’ interaction was significant (hits and RTs: all F<.74, all p>.40).

#### Learning dynamics: preliminary discussion (fig. 3)

In both the baseline and the convergent bias session, analyzing the evolution of response patterns over time (from time-step 1 to time-step 2) revealed an early preference for the optimal behaviors (see fig. 3a,b). This preference was already present in the first half of the baseline session and did not vary further with increasing probabilities. Interestingly, this preference for behaviors that minimized biomechanical costs seemed impervious to their probabilistic likelihood sampled from past observations. This suggests that biomechanical priors might short-circuit probabilistic sampling, and might interfere with decisions based on the extraction of statistical regularities.

In the divergent bias session (suboptimal bias), the evolution of the response pattern from time-step 1 to time-step 2 suggests that the absence of a difference between performance for optimal and suboptimal behaviors – although the latter were more frequently shown – could be primarily due to participants' initial preferences for optimal behaviors (see fig. 3a,b). This preference progressively decreased over time as the probability of observing suboptimal behaviors concomitantly increased. However, overall, this increase was not sufficient to compensate for the participants' initial lack of preference toward suboptimal behaviors.

Finally, it is noteworthy that the number of responses toward optimal versus suboptimal behaviors was overall greater in the incomplete, relative to the complete, action movies in both the baseline and the convergent bias sessions. This difference may account for the fact that the rate of hits for both the optimal and suboptimal behaviors was very high in the complete movie blocks. Therefore, the number of responses for optimal behaviors, and hence the difference between the two types of behavior, could not further increase due to a ‘ceiling’ effect. Alternatively, this difference may be accounted for by the fact that, in conditions of visual uncertainty, individuals tended to favor responses that were consistent with their prior expectations. Interestingly, this assumption is consistent with the finding that one's priors (here, an intrinsic preference for optimal behaviors) are primarily used to complement sensory uncertainty in order to allow decisions, and thus actions, to be made even in cases of noisy signals or sparse data [11], [16].

## Discussion

The aim of this study was to test how the biomechanical expectations conveyed by tool affordances interact with prior knowledge about tool function and use, and whether this interaction influences predictions about a demonstrator's intentions when using tools. Here, we provide the first evidence that low-level local cues such as object affordances influence the learning and prediction of tool-use behaviors. We demonstrate that biomechanical priors modulate the extent to which participants' predictions are influenced by probabilistically-induced prior expectations (see fig. 2). In particular, we found that when the demonstrator's behavior satisfied both the participants' biomechanical and probabilistic priors, the learning cost decreased, as participants efficiently combined both types of priors to make their predictions. Conversely, when the demonstrator's behavior violated the biomechanical but not the probabilistic priors, the learning cost increased, as participants had to deal with two conflicting sources of prior information.

Specifically, the dynamics of the integration of these probabilistic expectations was strongly dependent on the biomechanical optimality of the observed behaviors (see fig. 3). When the probabilistic bias favored suboptimal behaviors, participants needed a greater number of observations to neutralize a preference for optimal behaviors, as well as to derive and use probabilistic information to predict suboptimal behaviors. Furthermore, performance during both the baseline and the convergent bias sessions showed that participants exhibited an initial preference for optimal behaviors that was sustained throughout the session, and did not vary with changes in probabilistic bias. Interestingly, this initial preference was even stronger in the interrupted sequences, where subjects had little information about the demonstrator's intention. The strong influence of biomechanical priors in these sequences suggests that these priors might be primarily used in the case of noisy signals or sparse data. As such, they may be specifically suited to reduce the intrinsic uncertainty of goal-directed behaviors [16]. In sum, biomechanical priors provided by the tool's affordances acted as an inductive bias [13], complementing the available perceptual information when this information did not sufficiently constrain the number of potential solutions (e.g. ‘opening a box’ versus ‘switching the lights on’).

Together, these findings complement recent results published by Chambon and co-workers [11]. In their study, participants were requested to infer the intentions of a demonstrator who performed various actions on meaningless objects. The authors showed that as the amount of visual information conveyed by movement kinematics progressively decreased, participants responded more frequently toward the intentions that had the highest probability of occurring. Chambon et al.'s findings are consistent with a Bayesian estimation scheme: the less information one has about the action scene, the greater the weight of one's priors in the decision. Put another way: the higher the sensory uncertainty, the more the probabilistic bias is used to ‘resolve’, or ‘complement’, this sensory uncertainty. Our findings suggest that the effect of priors gathered from probabilistic sampling of past observations also depends on whether or not the visual information conveyed by the movement's kinematics meets the expectations that are induced by an object's affordances.

Even though visual information did not meet these expectations, participants tended to assume the demonstrator to behave in an optimal way. In other words, they expected the demonstrator to act as a ‘rational’ agent – i.e., an agent who adopts the most optimal (i.e., least costly) action means to achieve his goal given the constraints of the current situation. This echoes recent evidence showing that humans, even at a very early age, consider their conspecifics to be rational agents [8], [38], [39]. Thus, children may posit states of the world occasionally counterfactual to the perceptual evidence (such as the presence of occluded physical objects) but consistent with a rational interpretation of the observed action [40], [41]. Here, we show that, rather than being restricted to external, environmental aspects of reality (e.g., a ball jumps an obstacle to reach a new location versus a ball jumps to reach a new location but there is no obstacle present), the situational constraints through which the rational attributes of an observed behavior are estimated, are extended to self-centred, sensorimotor properties that observers share with the observed agents.

This issue is currently debated in the literature. On one hand, previous findings suggest that in early infancy such sensorimotor cues do not play an essential, selective role in the rational interpretation of observed actions. For example, Southgate and colleagues[42] showed that 6- to 8- month-old infants attributed rational properties to observed actions even when the movements used to achieve them were biomechanically impossible. In their study, rationality was defined as conditions in which the observed goal-directed movements were adapted to external situational constraints, independently of the biomechanical plausibility of these movements. On the other hand, other evidence suggests that a rational interpretation of goal-directed actions may be predicated upon sensorimotor information conveyed by movement kinematics [43]. On a similar line, Southgate and co-workers [44], [45] recently showed that the motor system of 9- to 15-months old infants was activated during the prediction of observed actions. The authors proposed that the activation of the motor system, instead of being driven by current visual information, was driven by the infants' expectations about the movements by which an attributed goal would likely be achieved. Given these contradicting data, one may speculate that the coupling of a rational interpretation of goal-directed actions with the processing of sensorimotor cues such as object affordances might be highly dependent on motor expertise acquired from experience [46]. Furthermore, this coupling might mature later in development. Our results suggest that the coupling of biomechanical with probabilistic priors may be particularly strong in adult observers, presumably equipped with a high degree of motor expertise.

Biomechanical and probabilistic priors may recruit two different – and parallel – neural systems that occasionally combine to derive information about tool use and function from observation. However, the exact nature and function of these systems is still a matter of conjecture. Effector-dependent, biomechanical priors may exert their influence on action prediction by differently weighting action alternatives within the motor repertoire of posterior frontal cortices such that certain actions become favored over others according to the biomechanical constraints of the motor effectors. This process of weighting action alternatives could be mediated by reciprocal inhibitory connections within the motor cortices, either by suppressing or increasing the activity of current competitors [47]. Occasionally, probabilistic priors may exert top-down influences on the selection of action alternatives within premotor cortices by using evidence gathered from past events to re-assigning new weights to the set of possible actions. Interestingly, these probabilistic priors may recruit more anterior frontal regions, such as the dorsolateral [48] or the inferior parts [49] of the dorsolateral prefrontal cortex. As a result, one may speculate that an abnormal connectivity between dorsolateral prefrontal and premotor regions – resulting from an impaired biasing influence from anterior to more posterior frontal cortices – would lead to abnormal action selection [50], [51]. Such abnormal selection might jeopardize acquisition of motor expertise and the ability to infer other people's intentions from observation [52].

### Conclusion

To our knowledge, the present study provides the first evidence that object affordances play a major role in the learning and prediction of observed tool-use behaviors. In particular, we show that perceiving observed behaviors as rational depends on low-level local cues from which their biomechanical costs are estimated with regard to their final goals. We suggest that biomechanical expectations elicited by affordances impede or bias the extraction of probabilistic regularities from past events. When these statistical regularities favor the observation of biomechanically suboptimal behaviors, biomechanical expectations delay the acquisition of probabilistic priors. Consequently, they also hinder the use of these priors in solving the uncertainty that is associated with incomplete visual signals.

Interestingly, one may extrapolate from our results that increasing the number of observations for suboptimal behaviors would further boost the weight devoted to probabilistic information in the participants' decisions. If this is the case what might this boost reflect and how might the brain represent it? Further studies should investigate how, and whether, the increasing weight of probabilistic information is associated with an update of biomechanical priors. Such an update could occur through a mechanism of visuomotor learning mediated by the plastic properties of the motor system [53]–[55]. This would allow one to determine whether the interaction between a ‘rational’ interpretation of actions and the detection of affordances recruits a modular, domain-specific process that would configure the experience of the external world *per se*. Implications for the social learning of tool use could be particularly important, as it would suggest that the larger the magnitude of this interaction for learners, the less able they would be to predict and learn from biomechanically suboptimal or unexpected behaviors. More generally, we believe that this cognitive selectivity for biomechanical optimality could contribute to the convergence of individual behaviors towards homogeneous patterns [17]. This could arise in the absence of high-level, faithful social transmission mechanisms such as true imitation of observed action goals and means [56]–[58]. Affordances could enhance the efficiency of less precise, though less costly, forms of social learning strategies in the acquisition of novel tool use, like emulation learning [59] or stimulus enhancement [60].
